# New benzene derivatives from cultures of ascomycete *Daldinia concentrica*

**DOI:** 10.1007/s13659-013-0048-1

**Published:** 2013-08-13

**Authors:** Tao Feng, Zheng-Hui Li, Xia Yin, Ze-Jun Dong, Gang-Qiang Wang, Xing-Yao Li, Yan Li, Ji-Kai Liu

**Affiliations:** 1 State Key Laboratory of Phytochemistry and Plant Resources in West China, Kunming Institute of Botany, Chinese Academy of Sciences, Kunming, 650201 China; 2 University of Chinese Academy of Sciences, Beijing, 100049 China; 3 School of Chemistry and Chemical Engineering of Hunan University, Changsha, 410082 China

**Keywords:** *Daldinia concentrica*, benzene derivatives, absolute configuration

## Abstract

Three new benzene derivatives, named daldins A-C (**1–3**), together with a known analogue, 2-hydroxymethyl-3-(1-hydroxypropyl) phenol (**4**) have been isolated from cultures of ascomycete *Daldinia concentrica*. The structures of **1–4** with absolute configuration were established by means of spectroscopic methods and X-ray diffraction. All compounds showed no significant inhibition on five human cancer cell lines with IC_50_ values > 40 μmol.

**Electronic Supplementary Material:**

Supplementary material is available for this article at 10.1007/s13659-013-0048-1 and is accessible for authorized users.

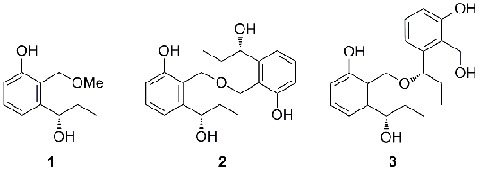

## Introduction

Ascomycete fungus *Daldinia concentrica* can be considered as a talent strain,[1] while a large number of natural products with diverse structures have been reported from both the fruiting bodies and fermentation broth. The initial chemical investigation of the fruiting bodies of *D. concentrica* reported two new 4:5:4′:5′-tetrahydroxy-1:1′-binaphthyl and dihydroxyperylene quinone in 1958,[2] more than 40 natural products have been so far isolated, including benzene derivatives,[3] azaphilone derivatives,[4] sesquiterpenoids,[5] squalene-type triterpenoids,[6] steroids,[7] cytochalasins,[3d,3e] etc. Of these, a substantial number possess significant bioactivities. For instance, concentricolide, a benzofuran lactone isolated from fruiting bodies of *D. concentrica*, exhibited the blockage ($EC_{50}$ 0.83 mg/mL) of syncytium formation between HIV-1 infected cells and normal cells.[3c] Additionally, the structure of concentricolide has been fully synthesized in 2011.[8] As our continuous search for novel secondary metabolites from higher fungi continued, we investigated the cultures of *D. concentrica*, which produced three new benzene derivatives, namely daldins A–C (**1**–**3**), together with a known analogue 2-hydroxymethyl-3-(1-hydroxypropyl)phenol (**4**).[9] In this paper we report the structure, elucidation and cytotoxicity of these isolates.

*To whom correspondence should be addressed. E-mail: jkliu@mail.kib.ac.cn

**1** R = Me
**4** R = H

**2**

**3**

## Results and Discussion

Compound **1** was isolated as colorless crystals (MeOH). The UV absorption at $\lambda_{max}$ 281 nm suggested the existence of a conjugated system. While the positive HRESIMS displayed an $[M + Na]^+$ peak at *m/z* 219.0993 (calcd. 219.0997 for $C_{11}H_{16}O_3Na$) indicating a molecular formula $C_{11}H_{16}O_3$. The $^1H$ NMR displayed three aromatic protons at $\delta_H$ 6.81 (1H, d, $J$ = 8.0 Hz, H-6), 6.95 (1H, d, $J$ = 8.0 Hz, H-4), and 7.18 (1H, t, $J$ = 8.0 Hz, H-5), corresponding to a 1,2,3-tri-substituted benzene ring. In addition, two methyls at $\delta_H$ 3.47 (3H, s, OMe) and 0.94 (3H, t, $J$ = 7.6 Hz, Me-10) were clearly identified.

**Table 1. NMR spectroscopic data for 1 and 2 ($\delta$ in ppm, *J* in Hz)**

| No. | **1**[a] $\delta_H$ | **1**[a] $\delta_C$ | **2**[b] $\delta_H$ | **2**[b] $\delta_C$ |
|---|---|---|---|---|
| 1 | | 156.9, s | | 156.1, s |
| 2 | | 119.6, s | | 120.7, s |
| 3 | | 142.8, s | | 144.9, s |
| 4 | 6.95, d (8.0) | 117.7, d | 6.98, d (8.0) | 118.0, d |
| 5 | 7.18, t (8.0) | 129.1, d | 7.16, t (8.0) | 129.6, d |
| 6 | 6.81, d (8.0) | 115.9, d | 6.76, d (8.0) | 114.8, d |
| 7a | 4.83, br. s | 69.1, t | 4.88, d (11.0) | 63.6, t |
| 7b | | | 4.71, d (11.0) | |
| 8 | 4.72, t (7.0) | 73.1, d | 4.68, t (6.8) | 71.9, d |
| 9 | 1.70, m | 30.9, t | 1.62, m | 30.9, t |
| 10 | 0.94, t (7.6) | 10.4, q | 0.86, t (7.4) | 10.5, q |
| OMe | 3.47, s | 58.2, q | | |

[a]Recorded in $CDCl_3$; [b]Recorded in $CDCl_3$ + methanol-$d_4$.

The $^{13}C$ NMR and DEPT spectra displayed eleven carbon resonances for two methyls (one oxygenated at $\delta_C$ 58.2), two methylenes (one oxygenated at $\delta_C$ 69.1), four methines, and three quaternary carbons. A methyl signal at $\delta_H$ 0.94 (3H, t, $J$ = 7.6 Hz, Me-10) displayed as a triplet, suggesting the existence of an ethyl fragment (-$CH_2$(9)-$CH_3$(10)). Moreover, one oxygenated proton at 4.72 (1H, t, $J$ = 7.0 Hz, H-8) showed key HMBC correlations to C-9, C-10, and one olefinic carbon ($\delta_C$ 142.8, s), indicating a hydroxypropyl connected to the benzene ring. These data provided information that compound **1** was closely related to the known compound 2-hydroxymethyl-3-(1-hydroxypropyl)phenol (**4**),[9] except for a methyl substituted to the hydroxymethyl in **4**, as supported by the HMBC correlation from $\delta_H$ 3.47 (3H, s, OMe) to $\delta_C$ 69.1 (t, C-7). Detailed analysis of other HMBC correlations (Figure 1) suggested that the other parts of **1** were the same to those of **4**. The Optical Rotation (OR) experiment of **1** gave a negative data $[\alpha]_D^{20}$ – 20.6 ($c$ 0.11, MeOH), which suggested the *S* form of C-8 in **1** by comparison with data reported in analogue annullatin E.[10] Further, the X-ray diffraction identified the absolute configuration of **1** as shown in Figure 1. Compound **1** was, therefore, identified as daldin A, as depicted. Furthermore, the negative OR data of **4** (measured data: $[\alpha]_D^{20}$ – 23.6 ($c$ 0.12, MeOH); reported data: $[\alpha]_D^{20}$ – 26 ($c$ 0.9, MeOH)[9]) suggested that the stereochemistry of C-8 in **4** should be the same to that of **1**. Therefore, compound **4** can be identified as ($S$)-2-hydroxymethyl-3-(1-hydroxypropyl)phenol.

**Figure 1.** Selected HMBC correlations (→) and the X-ray structure of **1**

Compound **2** was isolated as a colorless oil. Preliminary analysis of NMR data suggested that **2** possessed a structure closely related to that of **4**. However, the HRESIMS showed an [M + Na]$^+$ peak at $m/z$ 369.1675 (calcd for $C_{20}H_{26}O_5Na$, 369.1677), corresponding to a molecular formula $C_{20}H_{26}O_5$. This important information suggested that compound **2** might be a dimer of **4**, possessing two completely symmetrical units. Further evidence was detected from $^{13}C$ NMR spectrum, in which the signal of the oxymethylene was presented as a downfield shift at $\delta_C$ 63.6 (t, C-7) ($\delta_C$ 58.3 in **4**[9]), suggesting that the two units were connected according to the ether bond at C-7. Detailed analysis of NMR and MS data confirmed that the structure of **2** was a dimer derivative of **4**. The negative OR data ($[\alpha]_D^{20}$ – 18.4 ($c$ 0.26, MeOH)) also suggested the *S* form of C-8 in **2**. Compound **2** was, therefore identified as daldin B.

Compound **3** was also isolated as a colorless oil. The HRESIMS identified the molecular formula $C_{20}H_{26}O_5$ (measured: $m/z$ 369.1673; calcd for $C_{20}H_{26}O_5Na$, 369.1677), the same to that of **2**. The patterns of 1D NMR spectrum suggested that compound **3** was also a dimer of **4**. In the $^{13}C$ NMR spectrum, two significant downfield shifts at $\delta_C$ 64.4 (t, C-7) and 82.0 (d, C-8′) suggested that two units were attached with bond of C-7-O-C-8′, which was further supported by the HMBC correlations from $\delta_H$ 4.52 (2H, br. s, H-7) to C-8′ and from $\delta_H$ 4.50 (1H, t, $J$ = 7.2 Hz, H-8′) to C-7. Further analysis of 2D NMR data suggested that the other parts of two units in **3** were the same to those of **4**. Therefore, compound **3** was established as daldin C.

Compounds **1**–**4** were evaluated for their inhibitory activities on five human cancer cell lines using the MTT method as reported.[11] Unfortunately no compound exhibited significant cytotoxicity with $IC_{50}$ values > 40 μmol.

**Table 2. NMR spectroscopic data for 3 in $CDCl_3$ ($\delta$ in ppm, *J* in Hz)**

| No. | $\delta_H$ | $\delta_C$ | | $\delta_H$ | $\delta_C$ |
|---|---|---|---|---|---|
| 1 | | 157.1, s | 1′ | | 157.2, s |
| 2 | | 121.2, s | 2′ | | 124.1, s |
| 3 | | 142.4, s | 3′ | | 138.9, s |
| 4 | 6.85, d (8.0) | 118.8, d | 4′ | 6.85, d (8.0) | 119.9, d |
| 5 | 7.18, t (8.0) | 129.5, d | 5′ | 7.18, t (8.0) | 129.3, d |
| 6 | 6.83, d (8.0) | 116.5, d | 6′ | 6.83, d (8.0) | 116.5, d |
| 7 | 4.52, br. s | 64.4, t | 7′a | 5.01, d (12.3) | 58.6, t |
| | | | 7′b | 4.73, d (12.3) | |
| 8 | 4.45, t (6.8) | 74.1, d | 8′ | 4.50, t (7.2) | 82.0, d |
| 9 | 1.56, m | 30.7, t | 9′a | 1.81, m | 30.6, t |
| | | | 9′b | 1.64, m | |
| 10 | 0.79, t (7.2) | 10.8, q | 10′ | 0.86, t (7.4) | 10.6, q |

## Experimental Section

**General Experimental Procedures.** Optical Rotations (OR) were measured with a Horiba SEPA-300 polarimeter. Ultraviolet (UV) spectra were obtained using a Shimadzu UV-2401A spectrophotometer. Infrared (IR) spectra were obtained on a Bruker FT-IR Tensor 27 spectrometer using KBr pellets. 1D and 2D NMR spectra were run on a Bruker AV-400 MHz or a Bruker AV600 MHz spectrometer with tetramethylsilane (TMS) as an internal standard. Chemical shifts ($\delta$) were expressed in ppm with reference to solvent signals. Mass spectra were recorded on an API QSTAR Pulsar 1 spectrometer. Column chromatography (CC) was performed on silica gel (200–300 mesh, Qingdao Marine Chemical Co., Ltd., Qingdao, China). An APEX DUO (Bruker) instrument was used for the single crystal X-ray diffraction. An Agilent 1100 series instrument equipped with Agilent ZORBAX SB-C18 column (5 µm, 4.6 mm × 150 mm) was used for high-performance liquid chromatography (HPLC) analysis, and a semi-preparative Agilent ZORBAX SB-C18 column (5 µm, 9.4 mm × 150 mm) was used for the sample preparation. Fractions were monitored by thin layer chromatography (TLC) (GF 254, Qingdao Marine Chemical Co., Ltd., Qingdao, China), and spots were visualized by 10% $H_2SO_4$ in ethanol.

**Fungal Material and Cultivation Condition.** Strain (S 0318) was isolated from tissue culture of the fruiting bodies of *D. concentrica* collected at Laojunshan, Yunnan Province, China, in July 2003 and identified by Prof. Mu Zang, Kunming Institute of Botany, Chinese Academy of Sciences. A voucher specimen (HKAS 40992) was deposited at the herbarium of Kunming Institute of Botany, Chinese Academy of Sciences. The mycelial cultures were derived from tissue plugs. Culture PDA medium: potato (peeled), 200 g, glucose, 20 g, $KH_2PO_4$, 3 g, $MgSO_4$, 1.5 g, citric acid, 0.1 g, and thiamin hydrochloride, 10 mg, in 1 L of deionized $H_2O$. The pH was adjusted to 6.5 before autoclaving, and the fermentation was carried out on a shaker at 25 °C and 150 rpm for 20 days.

**Extraction and Isolation.** The culture broth (20 L) was extracted three times with EtOAc. The EtOAc lay was evaporated *in vacuo* to yield an extract (10 g). The latter was subjected to a silica gel column eluted with petroleum ether-acetone (1:0 to 0:1) to afford fractions1–6. Fraction 2 (1.2 g) was separated by silica gel CC (petroleum ether–$Me_2CO$, 10:1 $\rightarrow$ 5:1) to afford two subfractions a and b. Fraction a (240 mg) was separated by HPLC (acetonitrile–$H_2O$, 4:6 $\rightarrow$ 6:4, 10 mL/min in 30 mins) to yield **1** (4.5 mg), **2** (1.1 mg), **3** (1.8 mg), and **4** (48 mg).

**Daldin A (1):** colorless crystals (MeOH); $[\alpha]_D^{20}$ – 20.6 (*c* 0.11, MeOH); UV (MeOH) $\lambda_{max}$ (log $\varepsilon$) 281 (1.42), 202 (2.41) nm; IR (KBr) $\nu_{max}$ 3384, 3169, 2966, 1587, 1469, 1280, 1200, 987, 799 $cm^{-1}$; $^1H$ (400 MHz) and $^{13}C$ (100 MHz) NMR data ($CDCl_3$), see Table 1; positive ion HRESIMS *m/z* 219.0993 (calcd for $C_{11}H_{16}O_3Na$, 219.0997).

**Daldin B (2):** colorless oil; $[\alpha]_D^{20}$ – 18.4 (*c* 0.26, MeOH); UV (MeOH) $\lambda_{max}$ (log $\varepsilon$) 282 (1.48), 202 (2.61) nm; IR (KBr) $\nu_{max}$ 3386, 3146, 2963, 1590, 1469, 1272, 1014, 958, 793 $cm^{-1}$; $^1H$ (400 MHz) and $^{13}C$ (150 MHz) NMR data ($CDCl_3$ + methanol-$d_4$), see Table 1; positive ion HRESIMS *m/z* 369.1675 (calcd for $C_{20}H_{26}O_5Na$, 369.1677).

**Daldin C (3):** colorless oil; $[\alpha]_D^{20}$ – 16.0 (*c* 0.19, MeOH); UV (MeOH) $\lambda_{max}$ (log $\varepsilon$) 281 (1.65), 202 (2.33) nm; IR (KBr) $\nu_{max}$ 3387, 3175, 2966, 1588, 1468, 1280, 987, 799 $cm^{-1}$; $^1H$ (400 MHz) and $^{13}C$ (150 MHz) NMR data ($CDCl_3$), see Table 2; positive ion HRESIMS *m/z* 369.1673 (calcd for $C_{20}H_{26}O_5Na$, 369.1677).

**Crystallographic data of daldin A (1):** $C_{11}H_{16}O_3$, MW = 196.24; $a$ = 5.01570(10) Å, $b$ = 7.8428(2) Å, $c$ = 26.9837(6) Å, $\alpha$ = 90.00°, $\beta$ = 90.00°, $\gamma$ = 90.00°, $V$ = 1061.46(4) $Å^3$, $T$ = 100(2) K, space group $P$212121, $Z$ = 4, $\mu$(CuKα) = 0.720 $mm^{-1}$, 5136 reflections measured, 1825 independent reflections ($R_{int}$ = 0.0477). The final $R_1$ values were 0.0509 ($I > 2\sigma(I)$). The final $wR(F^2)$ values were 0.1400 ($I > 2\sigma(I)$). The final $R_1$ values were 0.0514 (all data). The final $wR(F^2)$ values were 0.1405 (all data). The goodness of fit on $F^2$ was 1.052. Flack parameter = 0.0(3). The Hooft parameter is – 0.07(13) for 645 Bijvoet pairs. The crystal structure of compound **1** was solved by direct method SHELXS-97 and expanded using the difference Fourier techniques, refined by the program SHLXL-97 and the full-matrix least-squares calculations. Crystallographic data for the structure of compound **1** have been deposited with the Cambridge Crystallographic Data Centre (deposition no. CCDC 951553). Copies of these data can be obtained free of charge via www.ccdc.cam.ac.uk.

## Electronic Supplementary Material

Supplementary material is available in the online version of this article at http://dx.doi.org/10.1007/s13659-013-0048-1 and is accessible for authorized users.

## Acknowledgments

This project was supported by the National Basic Research Program of China (973 Program, 2009CB522300), the National Natural Sciences Foundation of China (30830113, 81102346), and Youth Innovation Promotion Association CAS.

**Open Access** This article is distributed under the terms of the Creative Commons Attribution License which permits any use, distribution, and reproduction in any medium, provided the original author(s) and source are credited.

## Electronic supplementary material

Supplementary material, approximately 1.45 MB.

## References

[1] Dai Y C, Cui B K (2008). Mycosystema.

[2] Robbins W J, Kavanagh F, Hervey A (1947). Proc. Natl. Acad. Sci. U.S.A..

[1] Grabley S, Thiericke R, Zeeck A, Grabley S, Thiericke R (2000). The chemical screening approach. Drug discovery from nature.

[2] Allport D C, Bu’Lock J D (1958). J. Chem. Soc..

[CR5] Hahimoto T, Tahara S, Takaoka S, Tori M, Asakawa Y (1994). Chem. Pharm. Bull..

[CR6] Quang D N, Hashimoto T, Tanaka M, Baumgartner M, Stadler M, Asakawa Y (2002). J. Nat. Prod..

[CR7] Qin X D, Dong Z J, Liu J K, Yang L M, Wang R R, Zheng Y T, Lu Y, Wu Y S, Zheng Q T (2006). Helv. Chim. Acta.

[CR8] Qin X D, Dong Z J, Liu J K (2006). Helv. Chim. Acta.

[CR9] Shao H J, Qin X D, Dong Z J, Zhang H B, Liu J K (2008). J. Antibiot..

[4] Hashimota T, Tahara S, Takaoka S, Tori M, Asakawa Y (1994). Chem. Pharm. Bull..

[5] Qin X D, Shao H J, Dong Z J, Liu J K (2008). J. Antibiot..

[CR12] Stadler M, Baumgartner M, Grothe T, Muhlbauer A, Seip S, Wollweber H (2001). Phytochemistry.

[CR13] Quang D N, Hashimoto T, Tanaka M, Baumgartner M, Stadler M, Asakawa Y (2002). Phytochemistry.

[CR14] Qin X D, Liu J K (2004). J. Nat. Prod..

[CR15] Quang D N, Lam D M, Hanh N T H, Que D D (2013). Nat. Prod. Res..

[8] Chang C W, Chein R J (2011). J. Org. Chem..

[9] Weber D, Gorzalczany S, Martino V, Acevedo C, Sterner O, Anke T (2005). Z. Naturforsch..

[10] Asai T, Luo D, Obara Y, Taniguchi T, Monde K, Yamashita K, Oshima Y (2012). Tetrahedron Lett..

[CR19] Mosmann T (1983). J. Immunol. Methods.

[CR20] Feng T, Li Z H, Dong Z J, Su J, Li Y, Liu J K (2011). Nat. Prod. Bioprospect..

