# Supplementary material for: New benzene derivatives from cultures of ascomycete Daldinia concentrica
Source: Nat Prod Bioprospect. 2013 Aug 13;3(4):150–3. doi: 10.1007/s13659-013-0048-1 (PMC4131580; doi:10.1007/s13659-013-0048-1)

Electronic Supplementary Material

New benzene derivatives from cultures of ascomycete *Daldinia concentrica*

Tao FENG,[a] Zheng-Hui LI,[a] Xia YIN,[a,b] Ze-Jun DONG,[a] Gang-Qiang WANG,[c] Xing-Yao LI,[a] Yan LI,[a] and Ji-Kai LIU[a,]*

[a]State Key Laboratory of Phytochemistry and Plant Resources in West China, Kunming Institute of Botany, Chinese Academy of Sciences, Kunming 650201, China

[b]University of Chinese Academy of Sciences, Beijing 100049, China

[c]School of Chemistry and Chemical Engineering of Hunan University, Changsha 410082, China

Received 4 July 2013; Accepted 1 August 2013

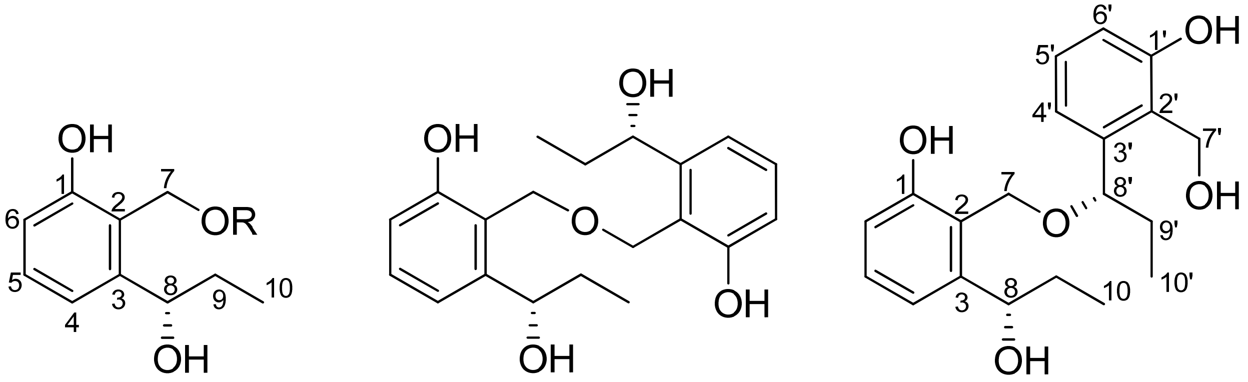

1 R = Me
4 R = H

2

3

Structures of compounds **1**–**4**

*To whom correspondence should be addressed. E-mail: jkliu@mail.kib.ac.cn

Contents

Figures 1S-5S: NMR and MS spectra of **1** ……S3-S7

Figures 6S-10S: NMR and MS spectra of **2**……S8-S12

Figures 11S-15S: NMR and MS spectra of **3**……S13-S17

Figures 16S-18S: NMR and MS spectra of **4** ……S18-S20

Figure 1S. ^{1}H-NMR spectrum of **1**.

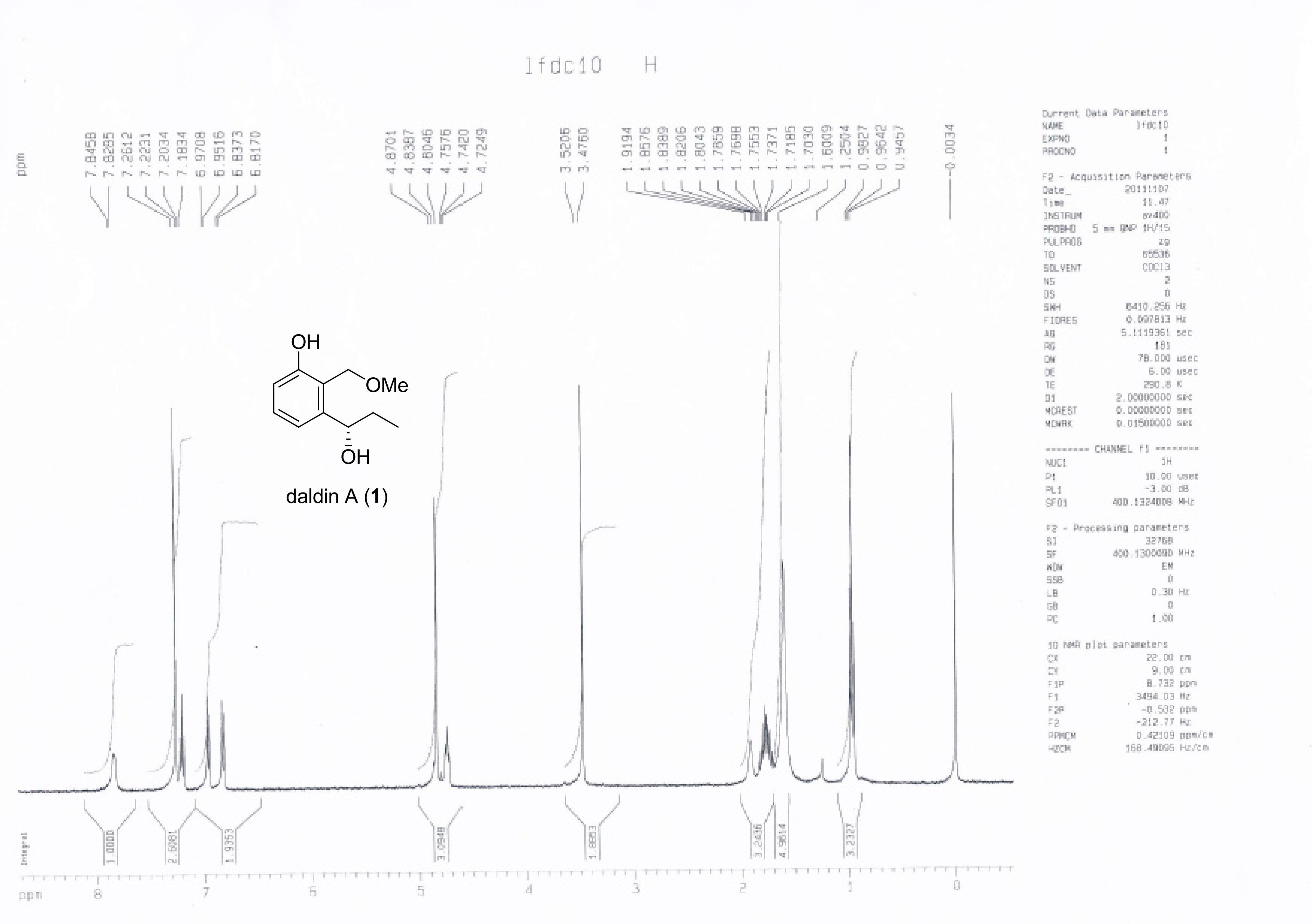

Figure 2S. ^{13}C-NMR spectrum of **1**.

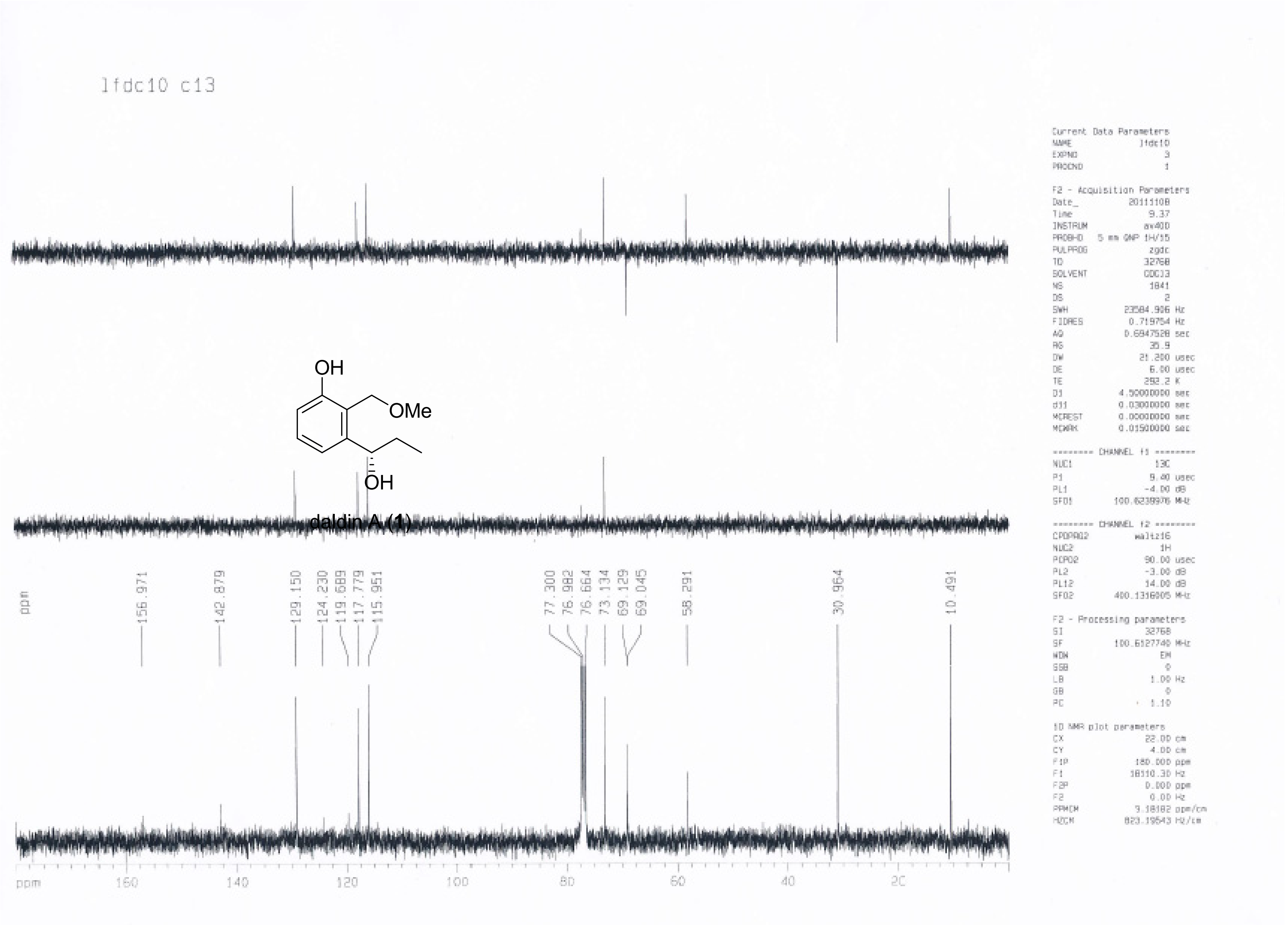

Figure 3S. HSQC spectrum of **1**.

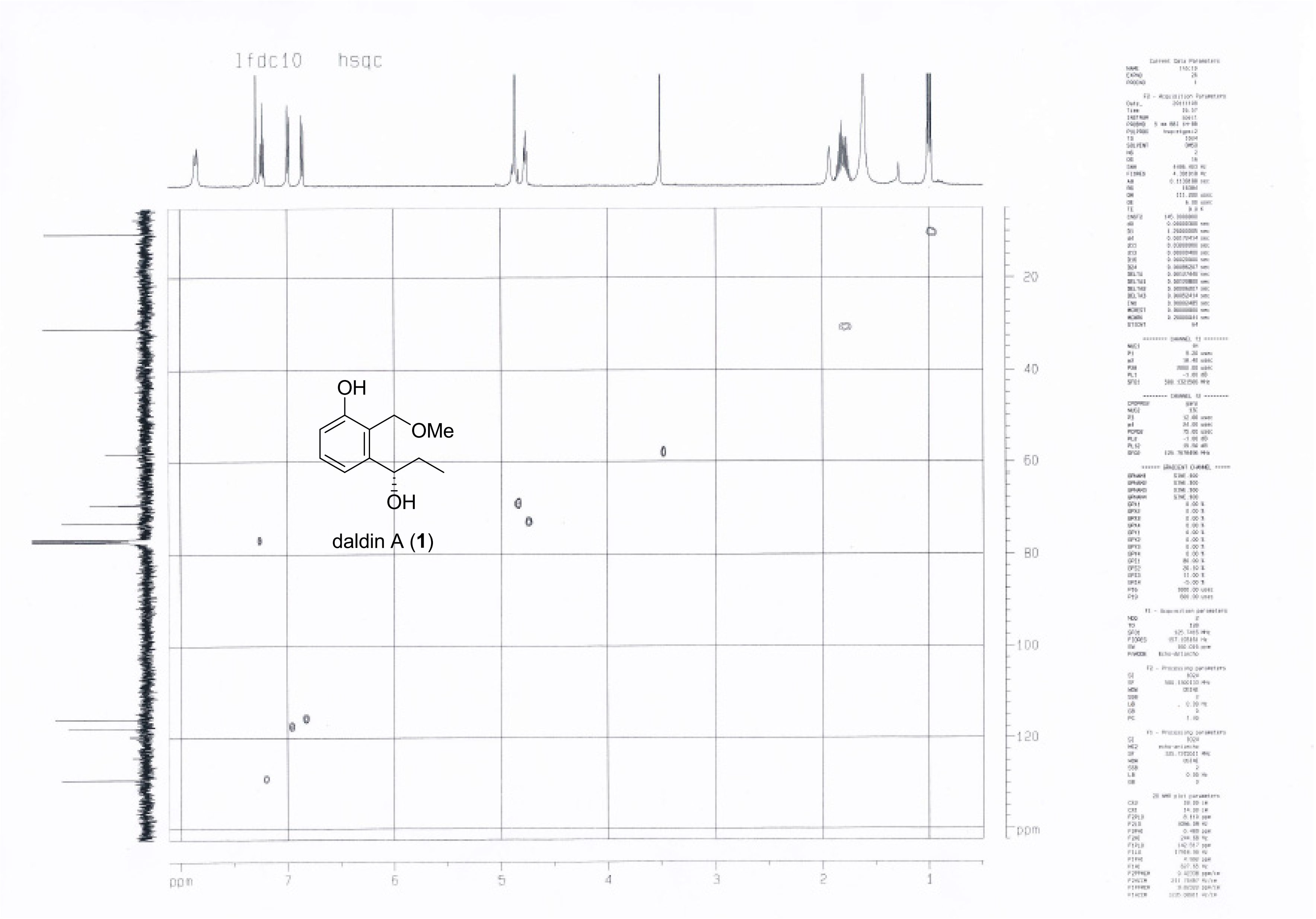

Figure 4S. HMBC spectrum of **1**.

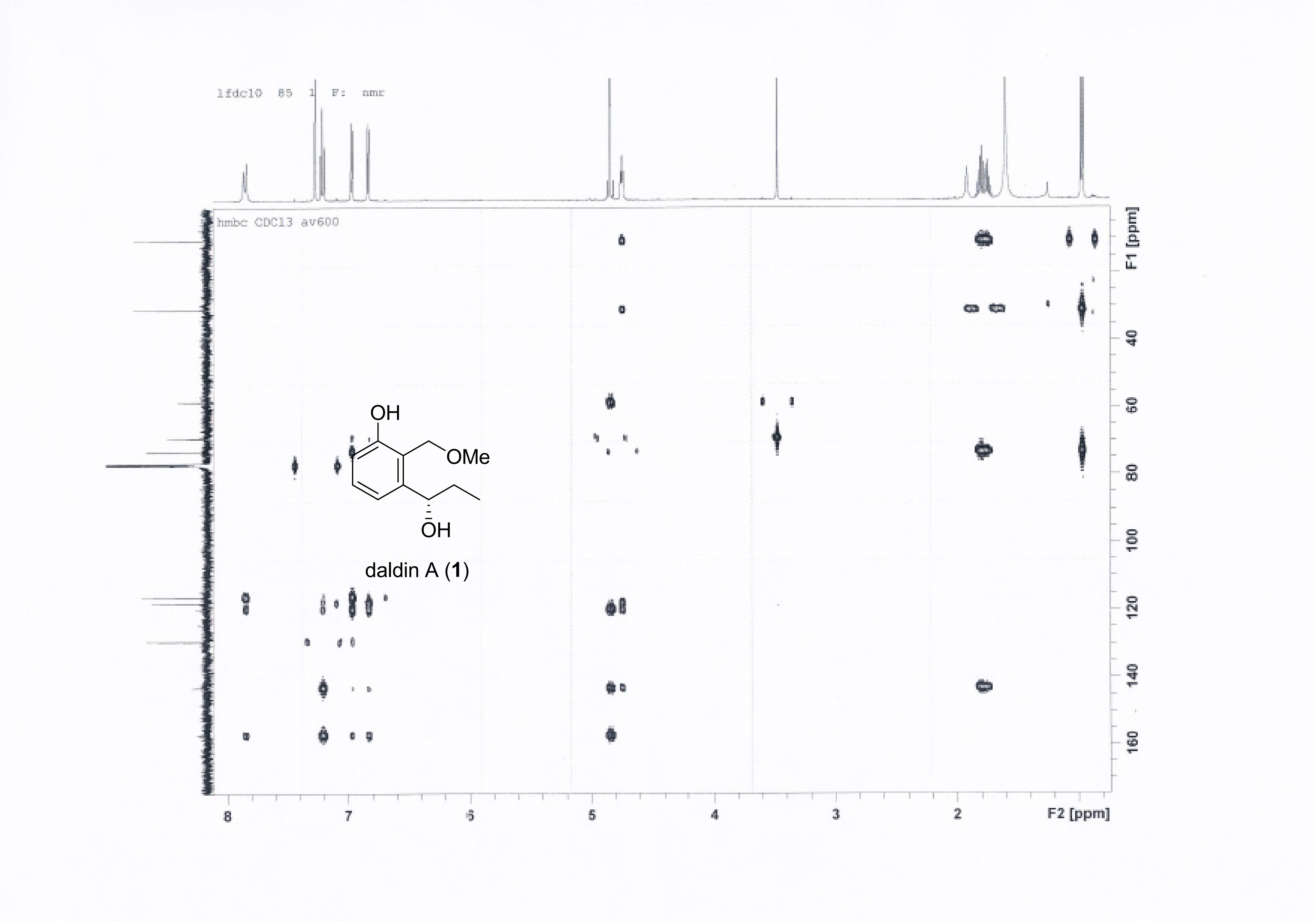

Figure 5S. HRESIMS of **1**.

Acq. Date: Wednesday, December 07, 2011 Acq. Time: 10:58

Sample Name: 111207ESIA 1fdc-10

Elemental composition calculator

Target m/z: +219.0993 amu
Tolerance: +10.0000 ppm
Result type: Elemental
Max num of results: 1000
Min DBE: -10.0000 Max DBE: +60.0000
Electron state: OddAndEven
Num of charges: 0
Add water: N/A
Add proton: N/A
File Name: 111207ESIA 1fdc-10.wiff

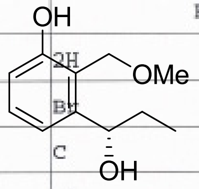

daldin A (**1**)

	Elements	Min Number	Max Number
1	2H	0	0
2	Br	0	0
3	C	0	200
4	Cl	0	0
5	F	0	0
6	H	0	400
7	I	0	0
8	K	0	0
9	N	0	0
10	Na	1	1

Figure 6S. ^{1}H-NMR spectrum of **2**.

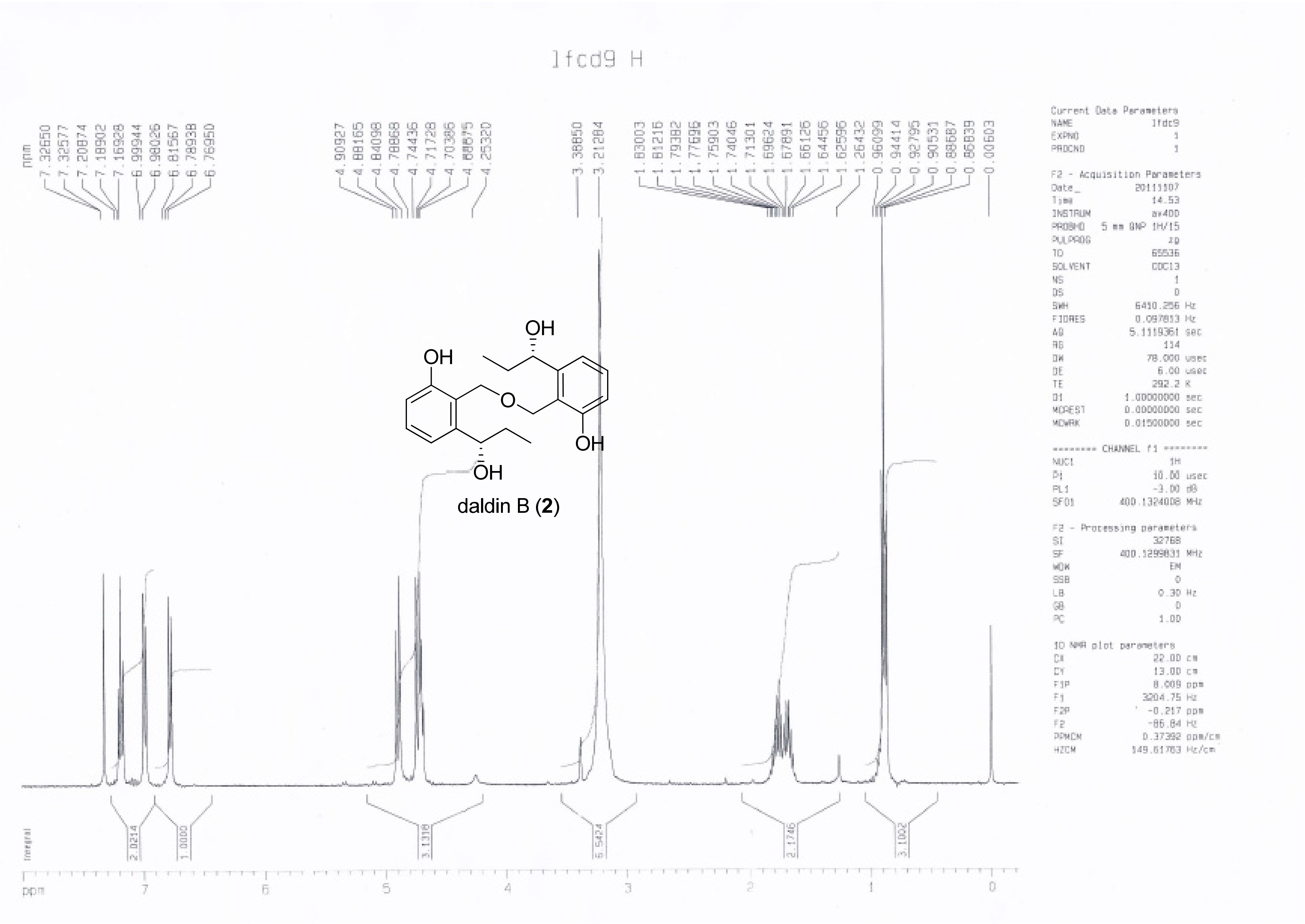

Figure 7S. ^{13}C-NMR spectrum of **2**.

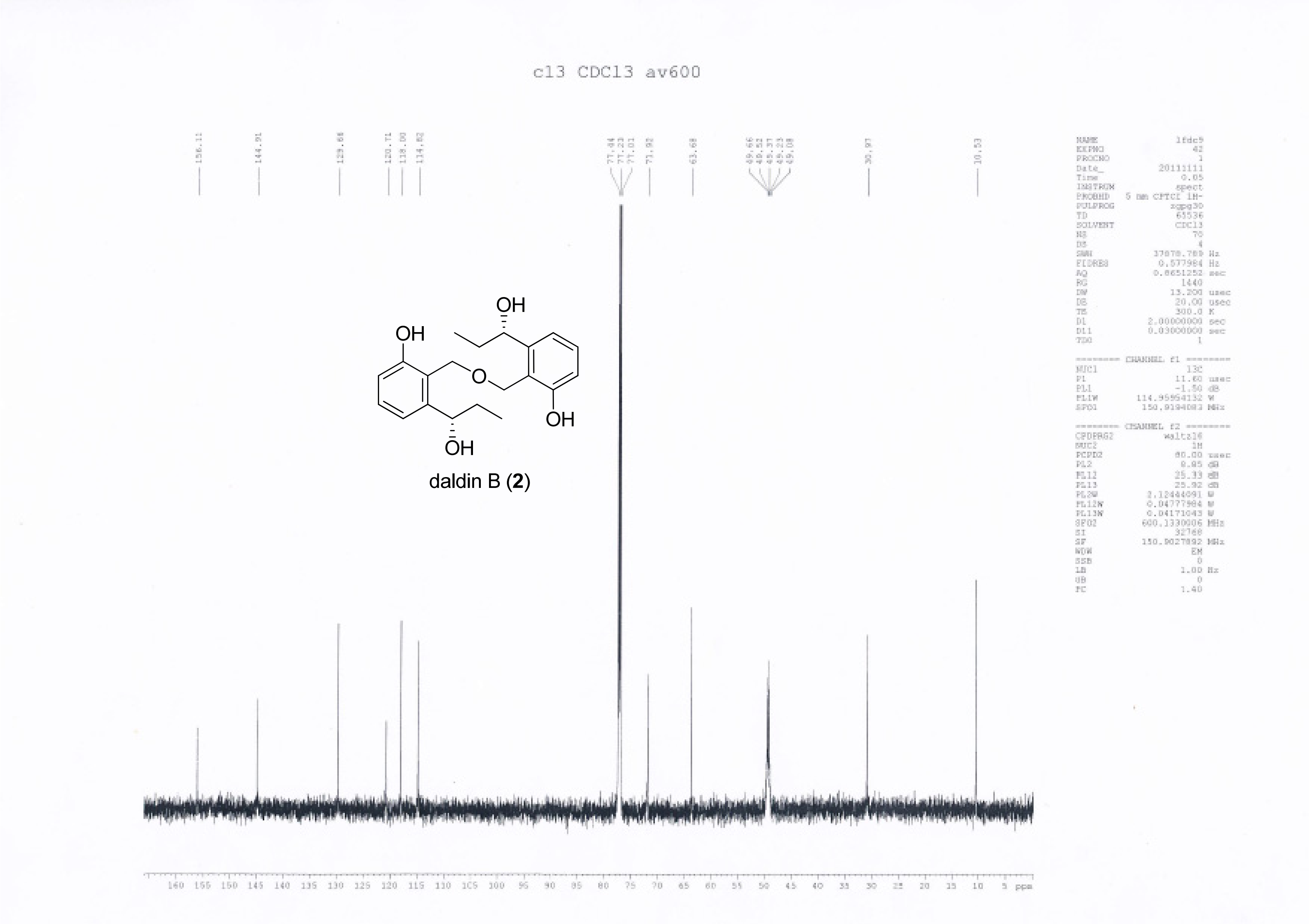

Figure 8S. HSQC spectrum of **2**.

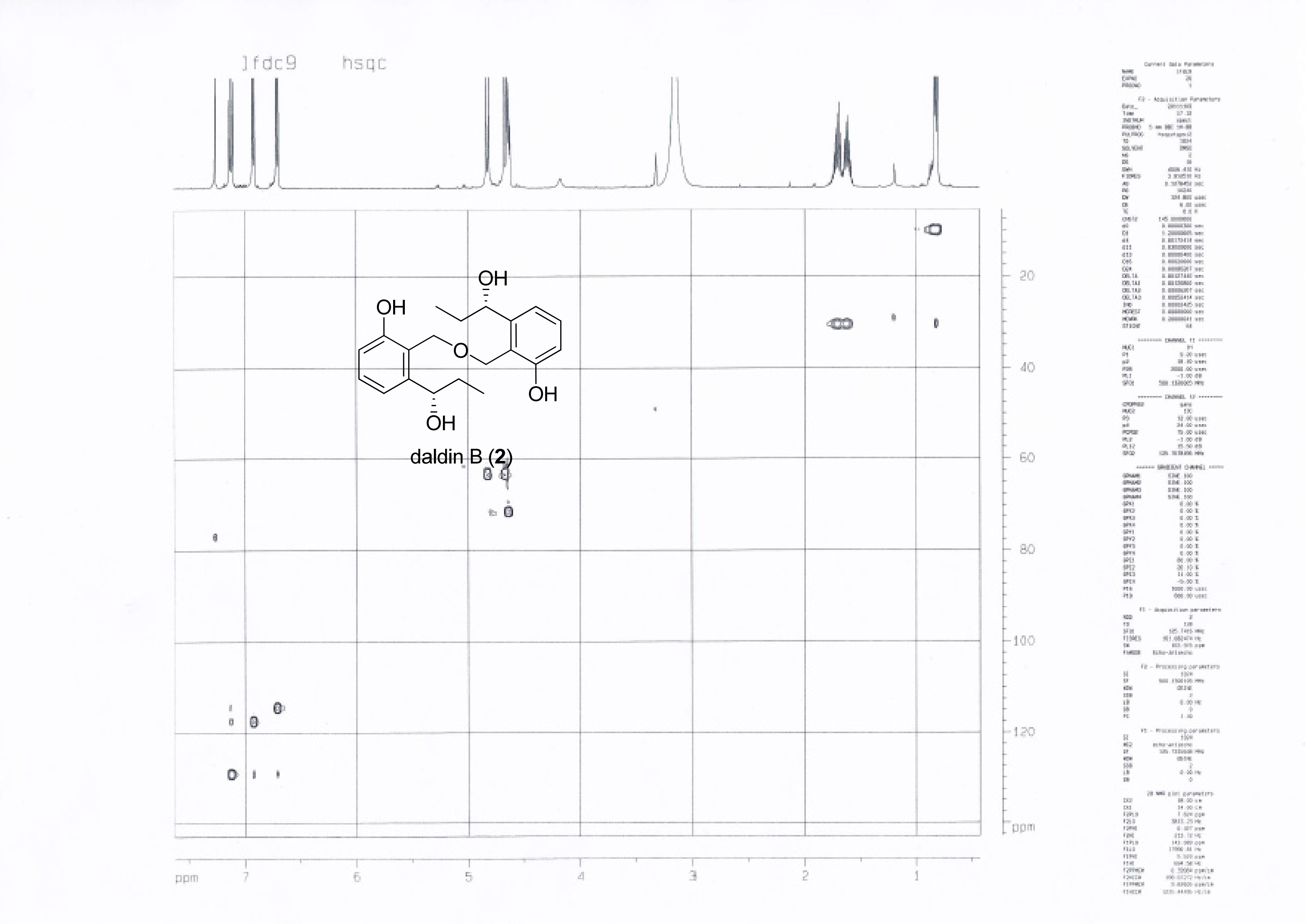

Figure 9S. HMBC spectrum of **2**.

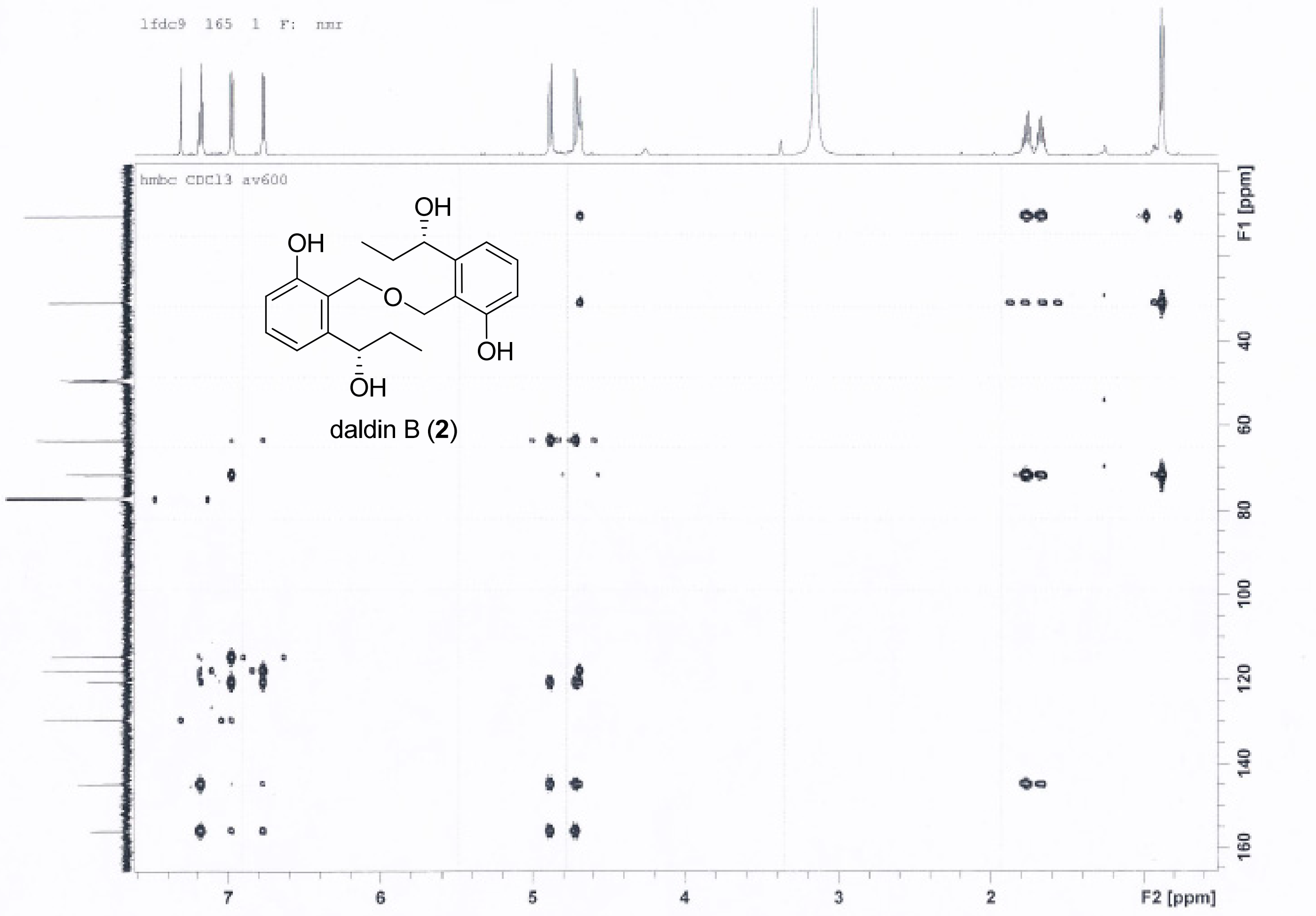

Figure 10S. HRESIMS of **2**.

Acq. Date: Wednesday, December 07, 2011   Acq. Time: 10:53

Sample Name: 111207ESIA   lfdc-9

Elemental composition calculator

Target m/z: +369.1675 amu

Tolerance: +10.0000 ppm

Result type: Elemental

Max num of results: 1000

Min DBE: -10.0000   Max DBE: +60.0000

Electron state: OddAndEven

Num of charges: 0

Add water: N/A

Add proton: N/A

File Name: 111207ESIA lfdc-9.wiff

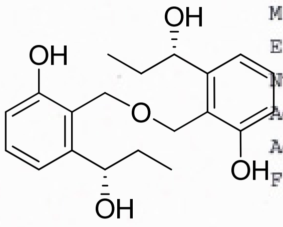

daldin B (**2**)

	Elements	Min Number	Max Number
1	2H	0	0
2	Br	0	0
3	C	0	200
4	Cl	0	0
5	F	0	0
6	H	0	400
7	I	0	0
8	K	0	0
9	N	0	0
10	Na	1	1

Figure 11S. ^{1}H-NMR spectrum of **3**.

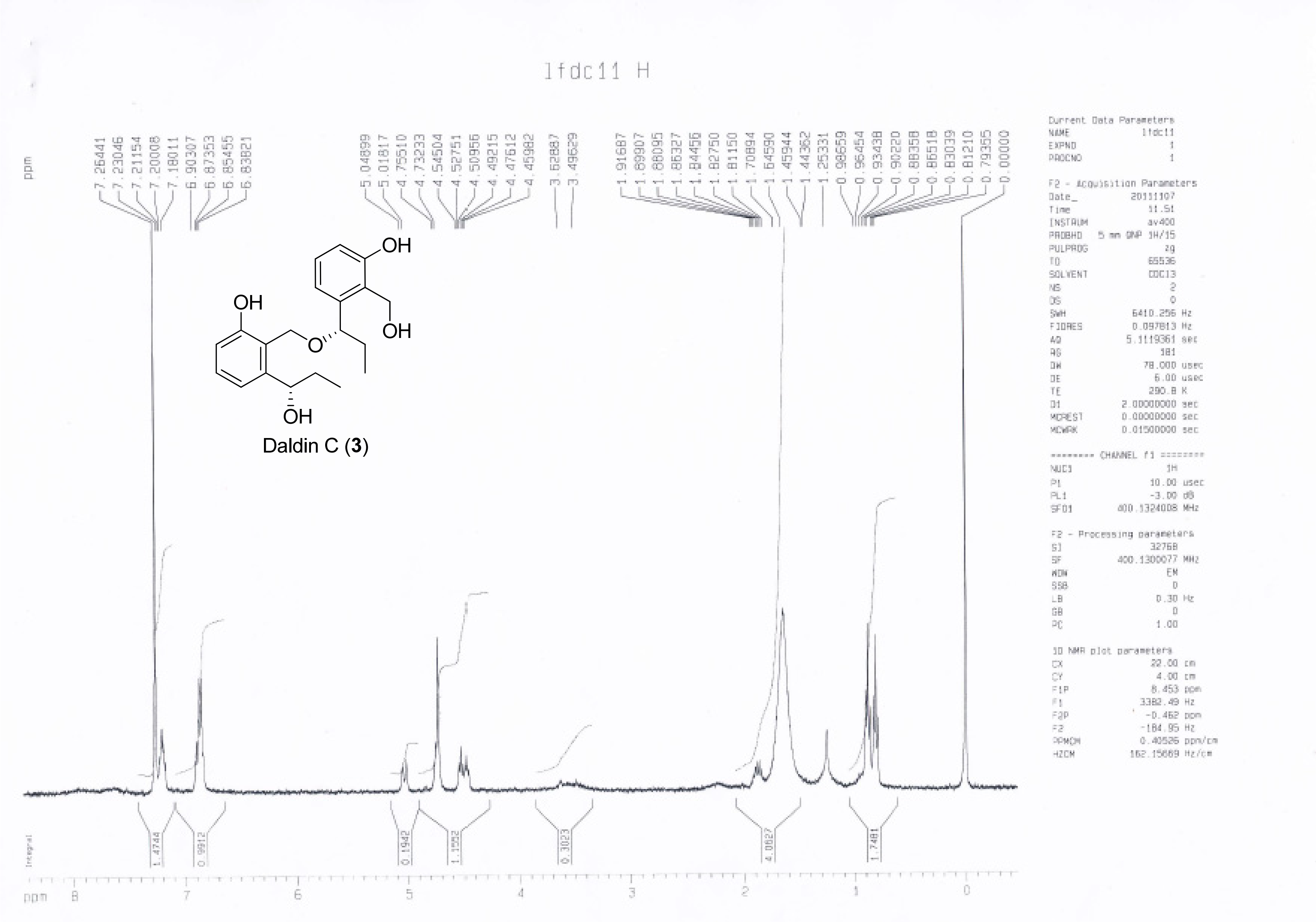

Figure 12S. ^{13}C-NMR spectrum of **3**.

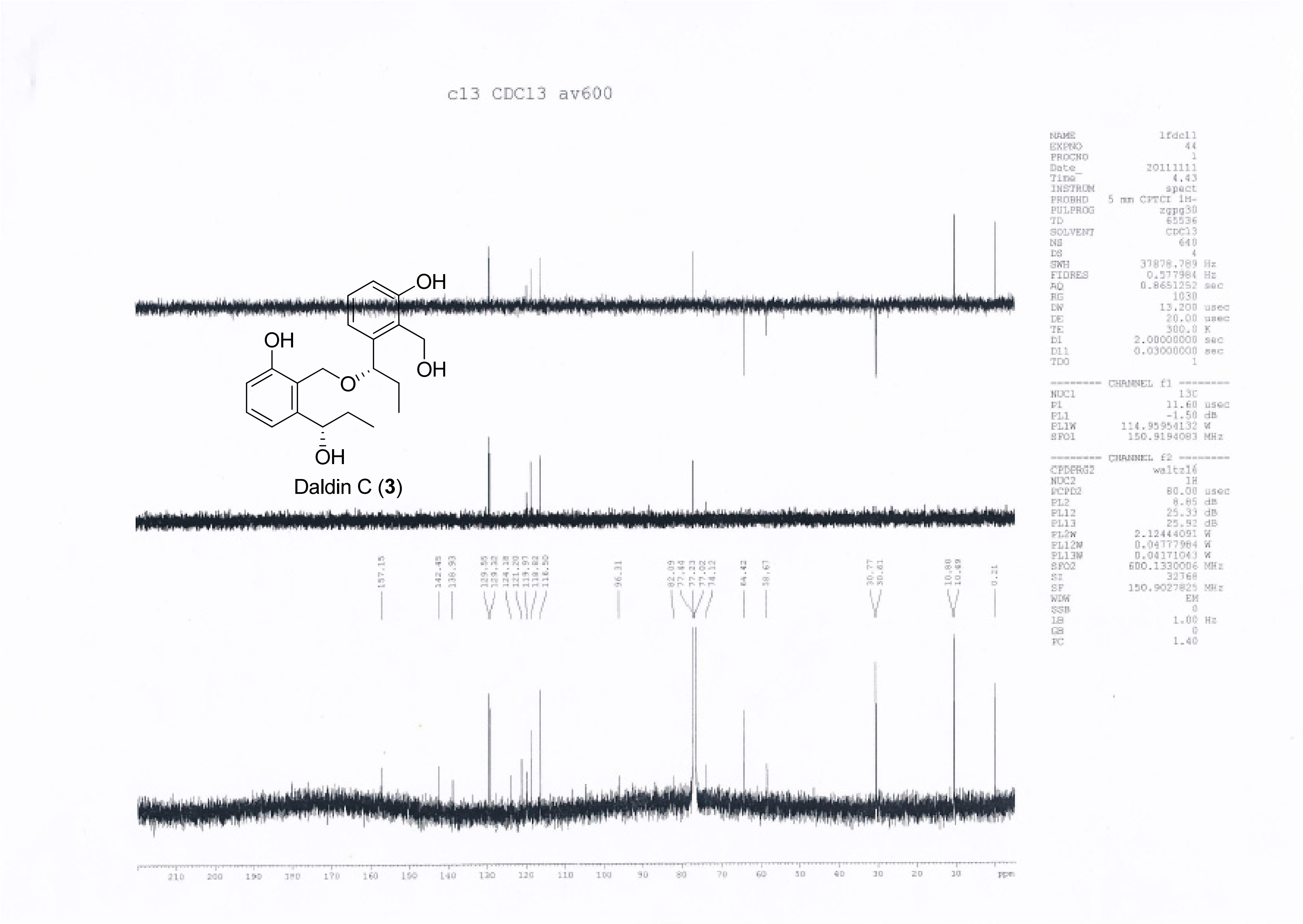

Figure 13S. HSQC spectrum of **3**.

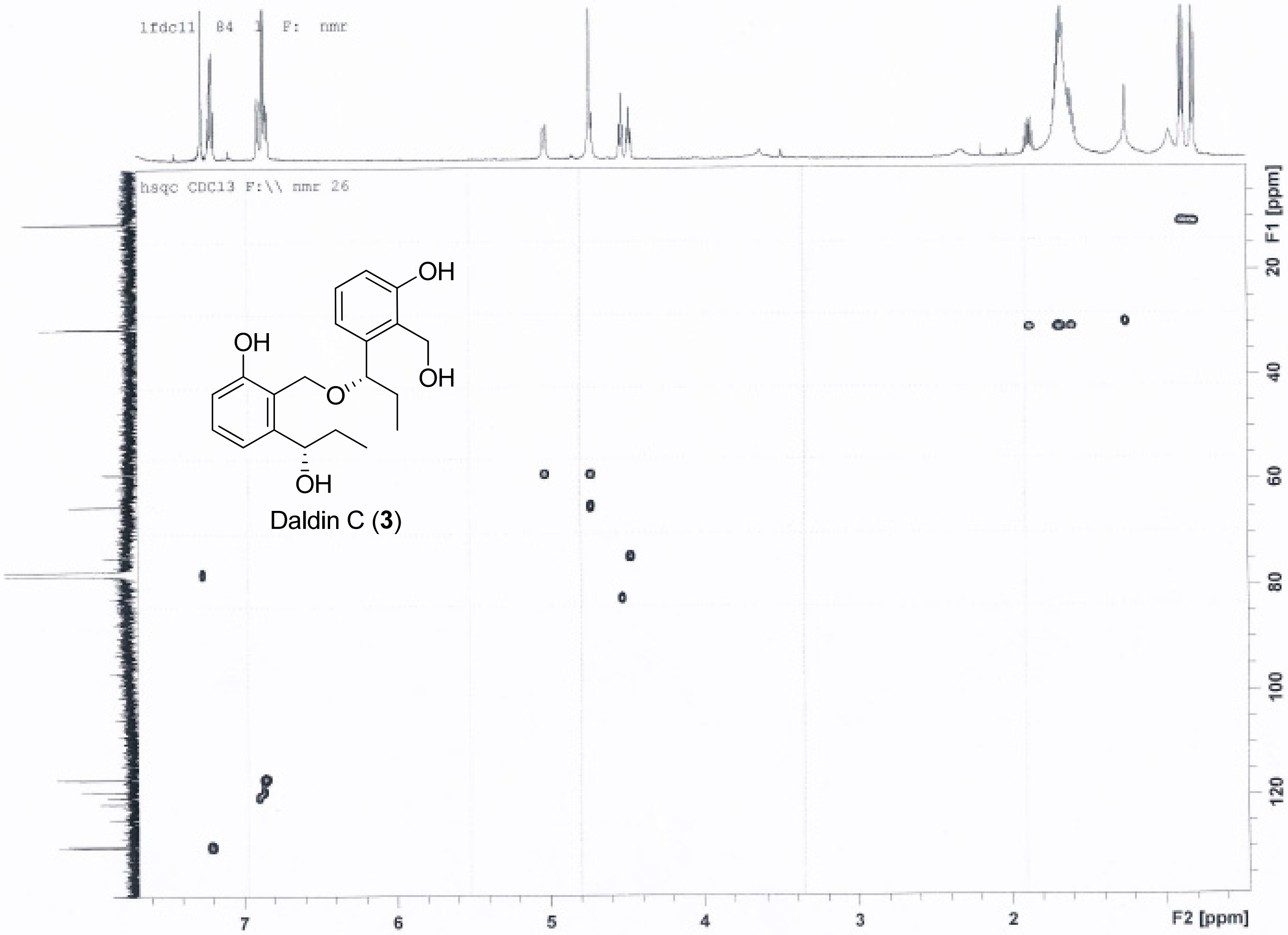

Figure 14S. HMBC spectrum of **3**.

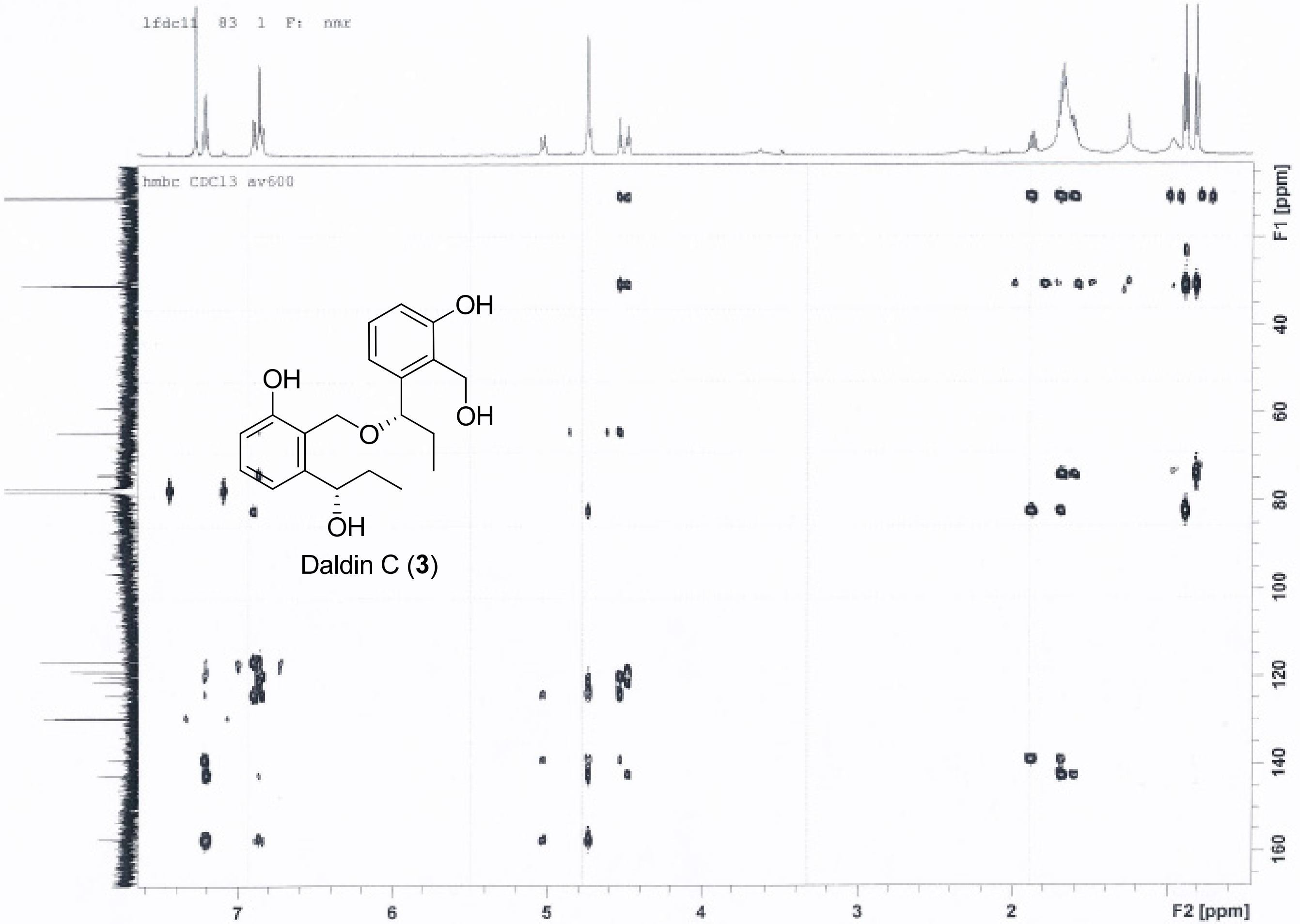

Figure 15S. HRESIMSof **3**.

Acq. Date: Wednesday, December 07, 2011   Acq. Time: 11:08

Sample Name: 111207ESIA lfdc-11

Elemental composition calculator

Target m/z: +369.1673 amu
Tolerance: +10.0000 ppm
Result type: Elemental
Max num of results: 1000
Min DBE: -10.0000 Max DBE: +60.0000
Electron state: OddAndEven
Num of charges: 0
Add water: N/A
Add proton: N/A
File Name: 111207ESIA lfdc-11.wiff

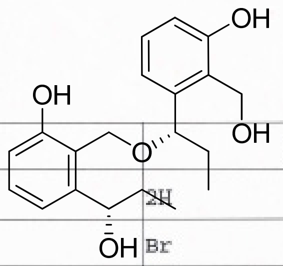

Daldin C (**3**)

	Elements	Min Number	Max Number
1	2H	0	0
2	Br	0	0
3	C	0	200
4	Cl	0	0
5	F	0	0
6	H	0	400
7	I	0	0
8	K	0	0
9	N	0	0
10	Na	1	1

Figure 16S. ^{1}H-NMR spectrum of **4**.

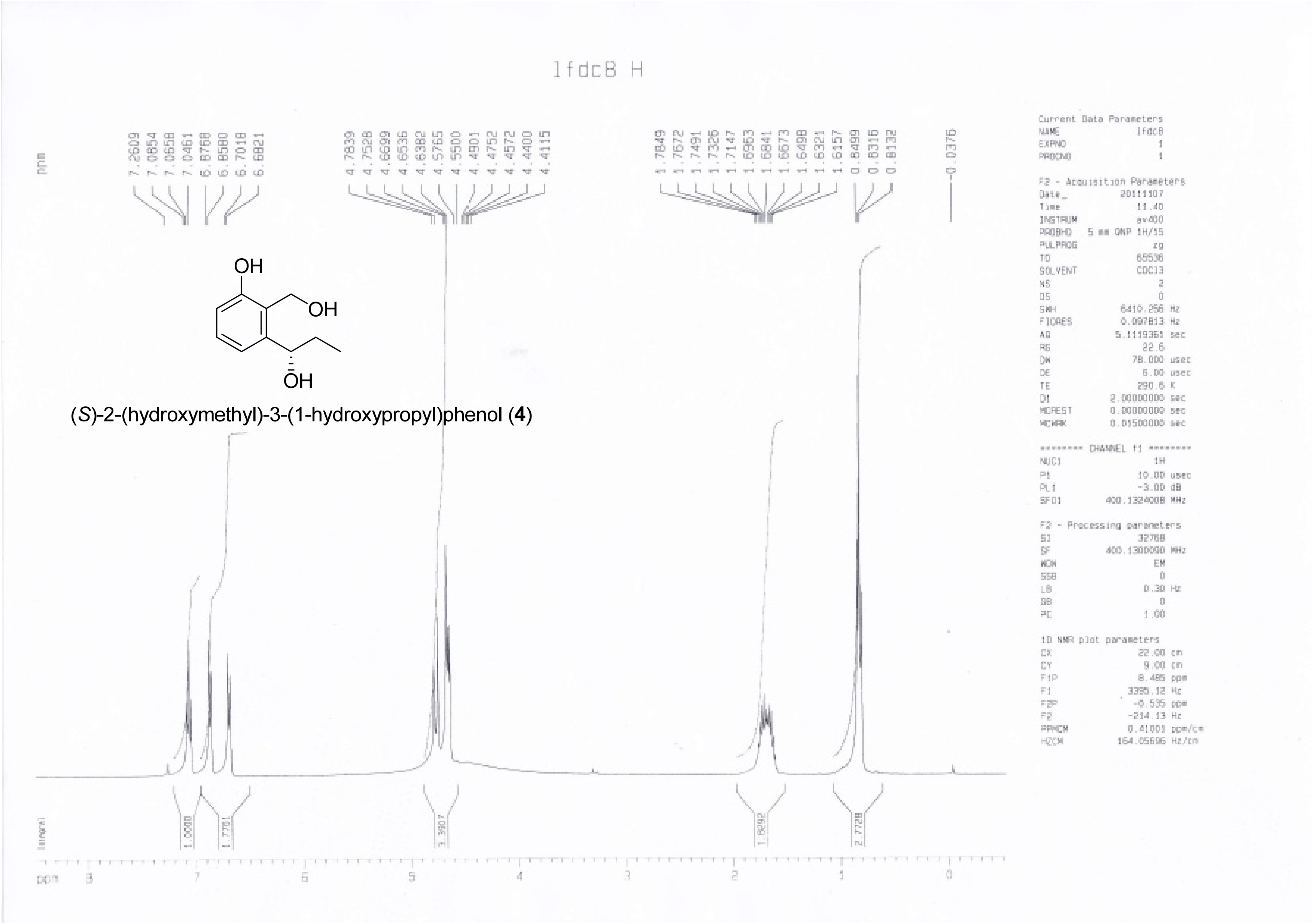

Figure 17S. ^{13}C-NMR spectrum of **4**.

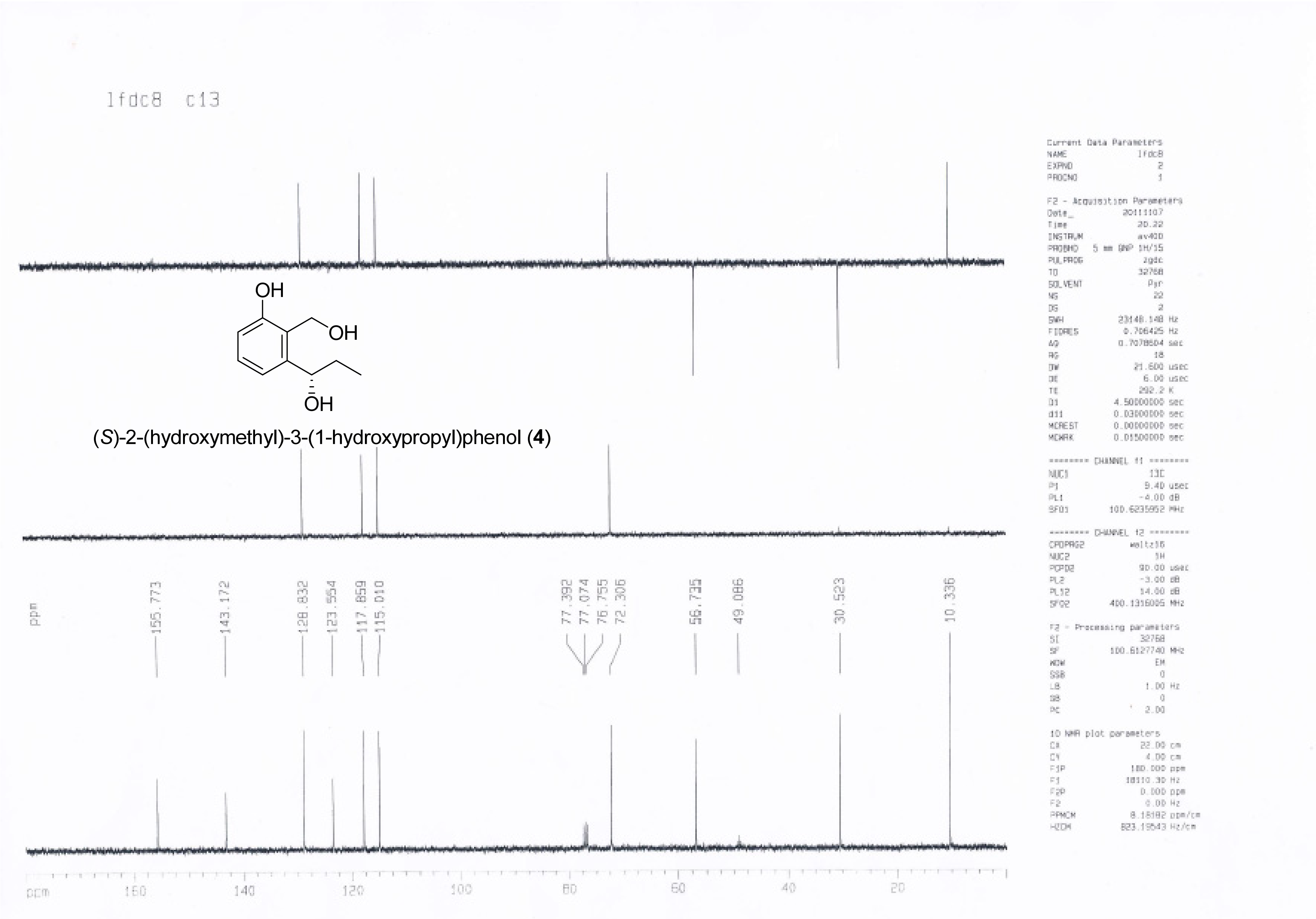

Figure 18S. ESIMS of **4**.

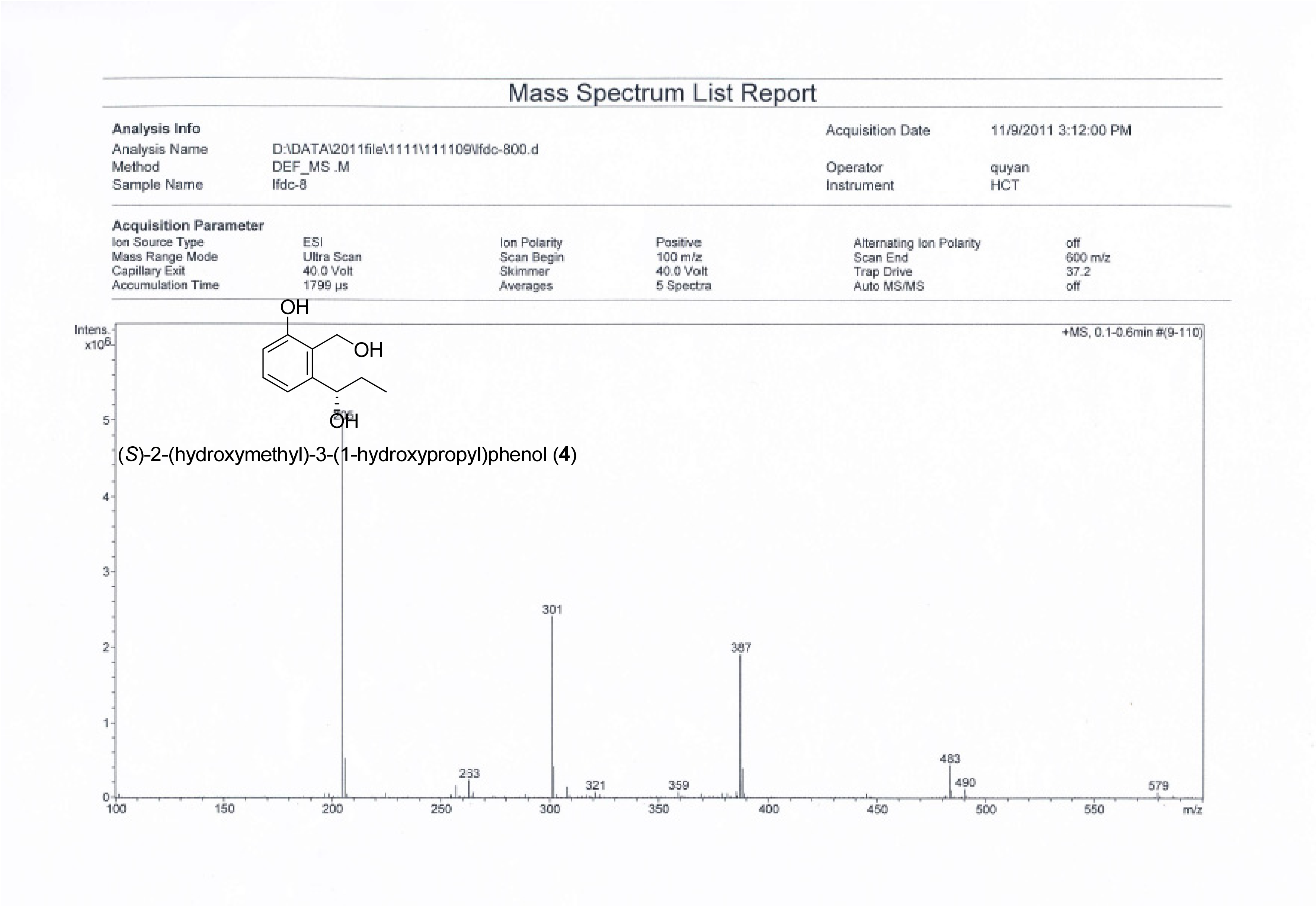

Supplement: Supplementary file 1 — Supplementary material, approximately 1.45 MB. [file 13659_2013_48_MOESM1_ESM.pdf]
